# Underlying Inborn Errors of Immunity in Patients With Evans Syndrome and Multilineage Cytopenias: A Single-Centre Analysis

**DOI:** 10.3389/fimmu.2022.869033

**Published:** 2022-05-17

**Authors:** Maurizio Miano, Daniela Guardo, Alice Grossi, Elena Palmisani, Francesca Fioredda, Paola Terranova, Enrico Cappelli, Michela Lupia, Monica Traverso, Gianluca Dell’Orso, Fabio Corsolini, Andrea Beccaria, Marina Lanciotti, Isabella Ceccherini, Carlo Dufour

**Affiliations:** ^1^ Hematology Unit, IRCCS Istituto Giannina Gaslini, Genoa, Italy; ^2^ Unità Operativa Semplice DIpartimentale (UOSD) Genetics and Genomics of Rare Diseases, IRCCS Istituto Giannina Gaslini, Genoa, Italy; ^3^ Pediatric Neurology and Muscular Diseases Unit, IRCCS Istituto Giannina Gaslini, University of Genoa, Genoa, Italy; ^4^ Stem Cell Transplantation Unit, IRCCS Istituto Giannina Gaslini, Genoa, Italy; ^5^ Laboratory of Molecular Genetics and Biobanks, IRCCS Istituto Giannina Gaslini, Genoa, Italy

**Keywords:** Evans syndrome, autoimmune cytopenias, inborn errors of immunity (IEI), immune dysregulation, autoimmune haemolytic anaemia (AIHA), ITP (idiopathic thrombocytopenic purpura), autoimmune neutropenia (AIN), ALPS (autoimmune lymphoproliferative syndrome)

## Abstract

**Background:**

Evans syndrome (ES) is a rare disorder classically defined as the simultaneous or sequential presence of autoimmune haemolytic anaemia and immune thrombocytopenia, but it has also been described as the presence of at least two autoimmune cytopenias. Recent reports have shown that ES is often a manifestation of an underlying inborn error of immunity (IEI) that can benefit from specific treatments.

**Aims:**

The aim of this study is to investigate the clinical and immunological characteristics and the underlying genetic background of a single-centre cohort of patients with ES.

**Methods:**

Data were obtained from a retrospective chart review of patients with a diagnosis of ES followed in our centre. Genetic studies were performed with NGS analysis of 315 genes related to both haematological and immunological disorders, in particular IEI.

**Results:**

Between 1985 and 2020, 40 patients (23 men, 17 women) with a median age at onset of 6 years (range 0–16) were studied. ES was concomitant and sequential in 18 (45%) and 22 (55%) patients, respectively. Nine of the 40 (8%) patients had a positive family history of autoimmunity. Other abnormal immunological features and signs of lymphoproliferation were present in 24/40 (60%) and 27/40 (67%) of cases, respectively. Seventeen out of 40 (42%) children fit the ALPS diagnostic criteria. The remaining 21 (42%) and 2 (5%) were classified as having an ALPS-like and an idiopathic disease, respectively. Eighteen patients (45%) were found to have an underlying genetic defect on genes *FAS*, *CASP10*, *TNFSF13B*, *LRBA*, *CTLA4*, *STAT3*, *IKBGK*, *CARD11*, *ADA2*, and *LIG4*. No significant differences were noted between patients with or without variant and between subjects with classical ES and the ones with other forms of multilineage cytopenias.

**Conclusions:**

This study shows that nearly half of patients with ES have a genetic background being in most cases secondary to IEI, and therefore, a molecular evaluation should be offered to all patients.

## Introduction

Evans syndrome (ES) is a rare disorder classically defined by the concomitant or sequential presence of autoimmune haemolytic anaemia (AIHA) and immune thrombocytopenia (ITP) (1–3), but it is also described as cytopenia due to the immune-mediated destruction of at least two blood cells lineages (4–6). It can be either idiopathic or secondary to other conditions, such as infections, inborn errors of immunity (IEI), autoimmune and rheumatologic diseases, malignancies, and drugs.

In paediatric age, IEI and in particular primary immuno-regulatory disorders (PIRDs) play a relevant role in the development of autoimmune cytopenias (7–9). In the first reported paediatric cohort of ES, about 10% of patients were identified as having IEI (10). Since then, the increased use of techniques like next-generation sequencing (NGS) and whole exome sequence (WES) revealed closer relationships between ES and PIRDS as autoimmune lymphoproliferative syndrome (ALPS), ALPS-like disorders, and common variable immunodeficiency (CVID) that often present with autoimmune cytopenias (AC) (10–13), and outlined the role of variants on the clinical phenotype of ES. This was clearly shown by two recent studies. The first found seven pathogenic variants on *CTLA4*, *LRBA*, *STAT3*, and *KRAS* genes in a cohort of 18 children with ES (14), whereas the second identified an underlying genetic defect in 65% of cases and showed that patients carrying variants displayed a more severe disease and required more lines of treatment versus the ones without (15). Similar findings came from another multicentre study on 60 children where underlying immune dysregulation was detected in 42% of cases (6). This is relevant because the detection of specific monogenic defects may not only address a correct diagnosis, but also enable the use of targeted therapies (4, 16–18).

However, in the above mentioned large, multicentre studies, genetic analysis was not offered to all eligible patients but was performed according to the request of the attending physicians. In addition, financial access to molecular analysis may have somehow affected the prevalence and the type of underlying disorders (6, 14, 15). Moreover, the issue of the potential genetic and immunological differences between classical ES (association of AIHA and ITP) and other forms of multilineage cytopenia was not addressed.

The aim of this study is to evaluate the genetic background and the clinical/immunological features of a single-centre cohort of paediatric patients with ES and other multilineage cytopenias.

## Materials and Methods

### Patients and Data

The clinical charts of all paediatric patients affected with classical ES and other multilineage cytopenias referred to our Unit between 1985 and 2020, identified *via* a clinical database, were reviewed.

AIHA was defined as the presence of haemolytic anaemia, a positive DAT, and the absence of other hereditary or acquired causes of haemolysis (16–18). AIN was defined as neutropenia due to the presence of indirect anti-neutrophil antibodies (19). ITP was defined as isolated thrombocytopenia (peripheral blood platelet count < 100,000 × 10^9^/l) in the absence of other causes or disorders that may be associated (20, 21).

Patients presenting with the association of AIHA and ITP with or without autoimmune neutropenia (AIN) were defined as having a classical ES. Children suffering from AIHA and AIN or ITP and AIN were considered as having a multilineage autoimmune cytopenia (MAC). ALPS was defined according to the revised diagnostic criteria by Oliveira et al. (22) which needs the presence of two required criteria in addition to a primary or secondary accessory criterion to state a definitive and probable diagnosis, respectively. Patients with both definitive and probable diagnoses were considered in the ALPS group of our cohort. Patients who did not completely fulfil the ALPS diagnosis but, in addition to cytopenia, presented with at least one required or primary additional criterion of ALPS diagnostic criteria were classified as having an ALPS-like disorder. Patients without diagnostic criteria of ALPS, ALPS-like, or any other underlying systemic disorder were considered as having a primary disease. Lymphoproliferation was defined as the presence of chronic (>6 months), non-malignant, non-infectious lymphadenopathy, hepatomegaly, or splenomegaly. Other immune abnormalities were defined as the presence of any of the following: inflammatory bowel diseases, autoimmune hepatitis, autoimmune thyroiditis, celiac disease, and auto-antibody positivity (ANA, ENA, ASMA, ASCA, ANCA, anti-ADAMTS13, anti-parietal, anti-dsDNA, anti-SSA/Ro, or LAC).

Data on demographics, clinical features, laboratory and immunological findings, management, and outcome were collected.

All adult subjects provided written informed consent to participate to this study, while parental consent was obtained for children, as approved by the Istituto Gaslini Ethical Committee. The study and all analyses conformed to the 1975 Declaration of Helsinki. Novel variants reported here for the first time have been submitted to ClinVar (https://www.ncbi.nlm.nih.gov/clinvar/) and assigned accession number SCV001424053-SCV001424130.

### Lymphocyte Immunophenotype and Serum Biomarkers

Peripheral lymphocyte subsets were evaluated from whole blood using an eight-colour immunostaining panel (lyse and wash procedure), a FACSCanto II flow cytometer (BD, Franklin Lakes, NJ, USA) equipped with three lasers (blue, red, violet), FACSDiva™ software (BD), and a large panel of RUO monoclonal antibodies and fluorochromes variously combined (all BD). CD3+CD4-CD8-TCRαβ+ T cells (double-negative T cells, DNTs) were calculated on total lymphocytes. An *in vitro* FAS-induced apoptosis test was considered pathological when positive in two separate assays.

### Genetic Analysis

DNA was isolated from peripheral blood samples of patients and their parents, when available, and tested for a selected list of genes through an NGS-based gene panel already reported (23). Sample library preparation and sequencing, and successive bioinformatics analyses, including variant annotation and interpretation, were carried out as described in Grossi et al. (23). The effect of variants was classified according with American College of Medical Genetics ACMG criteria (24) which are implemented in the VarSome database (www.varsome.com). In particular, the family segregation, population frequencies, functional prediction, and zygosity were taken into consideration.

Relevant variants were confirmed by polymerase chain reaction (PCR) amplification and direct Sanger sequencing of the corresponding DNA segments. X-inactivation analysis was performed using peripheral blood genomic DNA undigested or digested with restriction endonucleases sensitive to cytosine methylation (HpaII). PCR was carried out using two primers flanking the STR in the HUMARA gene. The PCR products were run on ABI PRISM 3130 (Applied Biosystems, Foster City, CA, USA). The ratio of active/inactive X chromosome was determined as described by Bolduc et al. (25).

### Statistical Analysis

Continuous variables were described as the median (range), and categorical variables were described as number (percentage). Quantitative variables were analysed using the χ^2^ test or Fisher exact test for small quantities. Differences between variables of various groups were considered significant when the P value was ≤ 0.05.

## Results

Forty patients (23 men, 17 women) with a median age at onset of 6 years (range 0–16) were studied. Twenty-two (55%) and 18/40 (45%) presented with ES and MAC, respectively. Seventeen out of 40 (43%) children met the ALPS diagnostic criteria. The remaining 18 (45%), 3 (7%), and 2 (5%) were classified as having an ALPS-like phenotype, CVID, and idiopathic cytopenia, respectively. Nine of the 40 (8%) patients had a positive family history of autoimmunity. Other immune abnormalities and signs of lymphoproliferation were present in 24/40 (60%) and 27/40 (67%) of cases, respectively. All patients but one required second- or further-line treatments which included mycophenolate mofetil (MMF) and sirolimus or both in 27 (67%), 18 (45%), and 14 (35%) cases, respectively.

The classical ES and MAC groups did not differ for any of the tested variables including family history of autoimmunity ALPS and ALPS-like/CVID phenotype with the exception of other immune abnormalities that were significantly more frequent (P = 0.03) in patients with MAC over those with classical ES. A trend of females to prevail in classical ES vs. MAC, without reaching statistical significance, was also observed **(** **Table 1** **).**

**Table 1:** Clinical and immunological differences according to type of cytopenia.

	ES n 22 (%)	MAC n 18 (%)	p
Presence of genetic variant	11/22 (50)	7/18 (39)	0.48
Females	12/22 (55)	5/18 (27)	0.08
Familiar history of autoimmunity	7/22 (32)	2/18 (11)	0.11
Associated immunological features^a^	10/22 (45)	14/18 (78)	0.03*
ALPS phenotype at onset^b^	10/22 (45)	7/18 (39)	0.67
ALPS-like/CVID phenotype at onset	10/22 (45)	11/18 (61)	0.32
Alive	20/22 (91)	18/18 (100)	0.18
DNT^c^> 1.5%	15/22 (68)	15/18 (83)	0.27
Need for second-line therapy	12/22 (55)	8/18 (44)	0.52
Sequential cytopenia	12/22 (55)	10/18 (56)	0.94

a Celiac disease, presence of autoantibodies including antinuclear, anti-neutrophils, anti-neutrophil cytoplasmic, anti-smooth muscle, anti-thyreoperoxydase, anti-thyroglobulin, lupus anticoagulant, anti-ADAMTS13, extractable nuclear antigen, cold agglutinins.

b Presence of required and accessory diagnostic criteria for ALPS.

c Double-negative T cells (CD3+ CD4- CD8- TCRαβ+).

*Statistically significant.

Genetic analysis was performed in all 40 patients, and variants were found in 18 (45%) of them. Variants were pathogenic/likely pathogenic in 13 and of unknown significance in 5 subjects. Patient carrying variants were not differently distributed in the classical ES and MAC groups. All variants defined as pathogenic were previously described or functionally validated (26, 27). Of note, they were all related to PIRDs and IEI. In particular, in 5/18 (28%) patients the gene was involved in the pathogenesis of ALPS (4 *FAS*, 1*CASP10*) and in 7/18 (39%) subject variants were implicated in ALPS-like disorders (4 *TNFSF13B*, 1 *LRBA*, 1 *CTLA4*, 1 *STAT3*). The remaining 6/18 cases (33%) were found to have an impairment in genes related to other IEI. Overall, genetic variants were found in 10/18 (55%) of patients with ALPS. **Tables 2**, **3** show the clinical/immunological details and the molecular results of patients carrying genetic variants, and the characteristics of the remaining cases are reported in **Table 4**.

**Table 2:** Clinical/immunological characteristics of patients carrying pathogenic/likely pathogenic variants related t o IEI (abnormal results in bold).

Pt, sex	Type of cytopenia	Clinical phenotype	Age (years) at diagnosis	Other clinical signs	Other abnormalities	Lymphocyte subpopulations					ALPS Cytokines			Immunoglobulin levels			Variant°	GnomADMAF	Varsome	SCT	Status
						L tot (/mmc)	T(%)	B(%)	NK (%)	DNT (%)	Il10 (pg/mL)	IL18(pg/mL)	Vit B12 (ng/L)	IgG (mg/dL)	IgA(mg/dL)	IgM(mg/dL)					
1, F	ITP, AIHA	ALPS	7	LPR	No	1,900	83	**6**	9	**2**	Na	Na	**1,581**	**Na**	**17**	81	*FAS* p.Glu256Gln	\	LP	No	Alive
2, M	ITP, AIHA	ALPS	6	LPR	No	1,757	78	15	**16.4**	**5.8**	**35**	**550**	921	**2,000**	100	43	*FAS* p.Glu 245Lys	\	P	No	Alive
3, F	ITP, AIHA	ALPS	1	LPR	No	**970**	75	**9.4**	**13.3**	**13**	**40**	**950**	**10,233**	1716	120	93	*FAS* p.Gln273His	\	P	No	Alive
4, F	ITP, AIHA	ALPS	12	LPR arthritis,	Anti-parietal ab	**510**	65	19	**13.5**	**2.2**	7	425	370	**600**	97	66	*CTLA4* p.Cys58Sfs*13	\	LP	No	Alive
5, F	ITP, AIHA, AIN	ALPS	2	LPR arthritis, sclerosing cholangitis	Anti TPO, anti-Tg, CD, ANA, LAC	**670**	87	**5.3**	2	**4.7**	0,2	265	999	**3,556**	199	244	IKBKG* p.Glu125Lys	0.00186	LP	No	Alive
6, F	ITP, AIHA, AIN	CVID	2	LPR	AI hepatitis, ANCA	4,125	62	20	**12.6**	**2.4**	0	**2,100**	0	1,185	119	14	*CARD11* p.Arg967Cys	0.0000294	VUS	No	Alive
7, F	ITP, AIHA, AIN	ALPS	5	LPR	No	5,060	97	**2.6**	1	**5.1**	14	**5,250**	374	**254**	**13**	299	*ADA2* p.Thr187Pro/ p.Leu188Pro *STAT3* p.Lys658Arg (mosaicisms)	/;0.00000882;/	LP/LP/LP	No	Dead
8, M	ITP, AIHA	Idiopathic	3	No	Recurrent infections	**315**	85	**0.2**	**14**	**1.7**	**385**	268	268	695	136	61	*LIG4* p.Arg278His HOMO	\	P	Yes	Alive
9, F	ITP, AIN	ALPS	1	LPR recurrent fevers	DAT; CD, ASMA, AI hepatitis	5,890	82	13	3.7	**2.6**	Na	Na	Na	955	54	67	*LRBA* Arg655Ter HOMO	0.0000147	P	No	Alive
10, F	ITP, AIN	ALPS	11	LPR recurrent fevers	No	1,690	73	19.9	1.1	**6**	**79**	**1,175**	**2,000**	1,689	293	110	*FAS* p.Cys129Arg	\	P	No	Alive
11, M	ITP, AIN	ALPS	9	LPR	CD	**60**	61	28	0	**3.5**	4	550	Na	Na	Na	Na	*STAT3^* p.Arg152Trp	\	LP	No	Alive
12, M	ITP, AIN	ALPS-like	2	No	AI hepatitis, ENA	**730**	**45.8**	38.2	**8.1**	**3**	Na	Na	**1,100**	**1,815**	26	**20**	*CARD11* p.Val1009Ile	0.000559	VUS	Yes	Alive
13, M	AIHA, AIN	ALPS-like	2	LPR, MAS	Cold agglutinins	**749**	**48**	15	4.1	0.4	Na	Na	400	**549**	**19**	**17**	*RAG1* p.Arg507Gln HOMO	\	LP	Yes	Alive

IEI, inborn errors of immunity; AI hepatitis, autoimmune hepatitis; AIHA, autoimmune haemolytic anaemia; AIN, autoimmune neutropenia; ANA, antinuclear antibodies; ANCA, anti-neutrophil cytoplasmic antibodies; ASMA, anti-smooth-muscle antibodies; B, benign; BMF, bone marrow failure; CD, celiac disease; DAT, direct antiglobulin test; DNT, double negative T cells; ENA, extractable nuclear antigen antibodies; ES, Evans syndrome; SCT stem cell transplant; ITP, immune thrombocytopenia; LAC, lupus anti-coagulant; LP, likely pathogenic; MAC, multilineage autoimmune cytopenia; MAS, macrophage activation syndrome; Na, not available; P, pathogenic; Tg, thyroglobulin; TPO, thyroid peroxidase; VUS, variant of unknown significance; LPR, lymphoproliferation (Chronic, > 6 months, non-malignant, non-infectious lymphadenopathy or splenomegaly or both). °Variant zygosity is always meant as heterozygosity unless differently reported * A ratio of active/inactive × chromosome equal to 70:30, demonstrating that a moderate skewing was detected. This finding suggests a correlation between skewed × inactivation and phenotype in carriers of X-linked disease. ^ Gain of function (28).

**Table 3:** Clinical/immunological characteristics of patients carrying pathogenic or of unknown significance variants related to risk factors for immune-dysregulation (abnormal results in bold).

Pt,sex	Type of cytopenia	Clinical phenotype	Age (years)at diagnosis	Other clinical signs	Other abnormalities	Lymphocyte subpopulations					ALPS cytokines			Immunoglobulin levels			Variant	GnomAD MAF	Varsome	SCT	Status
						L tot (/mmc)	T (%)	B (%)	NK(%)	DNT (%)	Il 10 (pg/mL)	IL18(pg/mL)	Vit B12 (ng/L)	IgG (mg/dL)	IgA(mg/dL)	IgM(mg/dL)					
1, M	ITP, AIHA	ALPS-like	4	No	No	2,298	**53**	**44**	0.6	0.4	Na	Na	732	1,197	52	106	*TNFRSF13B* p. Ser194Ter	\	P	No	Alive
2, F	ITP, AIHA, AIN	CVID	3	LPR, arthritis, recurrent fevers	CD, ANA	1,129	72	11	**15**	5	0,9	**1,225**	807	**250**	**15**	101	*TNFRSF13B* p.Arg202His	0,000729	VUS	No	Alive
3, M	ITP, AIHA	ALPS-like	1	No	No	4,375	57	**31**	**9.5**	**2.7**	11,2	**800**	1,005	**1,785**	84	157	*TNFRSF13B* p.Gln57His	0,00019	VUS	No	Alive
4, F	ITP, AIN	CVID	1	Congenital malformations hydrocephalous BMF	No	**310**	**52**	35	0.5	0.8	Na	Na	Na	Na	Na	Na	*TNFRSF13B* p.Arg202His	0,000729	VUS	Yes	Alive
5, F	AIHA, AIN	ALPS	1	LPR	DAT, ASMA	3,045	74	**9**	1.6	**4.1**	**90**	**5,001**	**1,492**	**1,596**	119	69	*CASP10* p.Val410Ile	0,0419	B°	Yes	Alive

AIHA, autoimmune haemolytic anaemia; AIN, autoimmune neutropenia; ANA, antinuclear antibodies; ANCA, anti-neutrophil cytoplasmic antibodies; ASMA, anti-smooth-muscle antibodies; B, benign; BMF, bone marrow failure; CD, celiac disease; DAT, direct antiglobulin test; DNT, double-negative T cells; ENA, extractable nuclear antigen antibodies; ES, Evans syndrome; SCT stem cell transplant; ITP, immune thrombocytopenia; LAC, lupus anticoagulant; LP, likely pathogenic; MAC, multilineage autoimmune cytopenia; MAS, macrophage activation syndrome; Na, not available; P, pathogenic; Tg, thyroglobulin; TPO, thyroid peroxidase; VUS, variant of unknown significance; LPR, lymphoproliferation (chronic, > 6 months, non-malignant, non-infectious lymphadenopathy or splenomegaly or both). °Abnormal functional test (29).

**Table 4:** Clinical/immunological characteristics of patients without genetic variant (abnormal results in bold).

Pt, sex	Type of cytopenia	Clinical phenotype	Age (years) at diagnosis	Other clinical signs	Other abnormal immunological features	Lymphocyte subpopulations					ALPS cytokines			Immunoglobulin levels			Status
						L tot(/mmc)	T(%)	B (%)	NK(%)	DNT (%)	IL 10 (pg/mL)	IL18(pg/mL)	Vit B12 (ng/L)	IgG(mg/dL)	IgA(mg/dL)	IgM(mg/dL)	
1, M	ITP, AIHA, AIN	ALPS	1,5	LPR	ANA	**690**	69	20	10.1	**15**	0	**550**	684	1,616	91	212	Alive
2, F	ITP, AIHA	ALPS-LIKE	6,8	LPR	No	**490**	87	**6**	**5**	1	0.9	**925**	378	834	105	50	Dead
3, F	ITP, AIHA	ALPS-LIKE	11,6	TTP	ANA	2,215	63	16	18	1	0	0	0	904	176	101	Alive
4, F	ITP, AIHA	ALPS-LIKE	0,3	No	No	1,785	81	**0.2**	**6**	**3**	1.3	280	361	839	13	4	Alive
5, M	ITP, AIHA	/	16,6	No	No	2,900	88	**3**	**0**	0.3	0	0	0	1,275	301	400	Alive
6, F	ITP, AIHA, AIN	ALPS-LIKE	5,3	LPR	ANA, ASMA	4,190	87	**1**	11	0.6	0	0	0	1,213	519	232	Alive
7, M	ITP, AIHA	ALPS-LIKE	6,8	No	ANA, LAC, ANCA, ENA, anti-dsDNA ab	**750**	67	17	14	3	13.2	375	0	546	62	25	Alive
8, M	ITP, AIHA	ALPS	11,6	LPR	Erythema nodosum	**1,050**	76	16	**0**	2	3	**2,000**	0	467	47	17	Alive
9, F	ITP, AIHA	ALPS	10,3	LPR	ANA, ENA, anti-dsDNA, anti-C3d, LAC, anti-Ro/SSA	1,270	84	10	**5**	3	0	**705**	283	1,412	163	107	Alive
10, M	ITP, AIN AIHA	ALPS-LIKE	15,3	No	ANA	1,580	83	**7**	10	3	0	0	0	1,229	220	67	Alive
11, F	ITP, AIHA	ALPS-LIKE	0,3	LPR	No	Na	Na	Na	Na	3	0	0	927	Na	Na	Na	Alive
12, M	ITP, AIN	ALPS-LIKE	8,8	No	Anti-N ab, anti-TG, anti TPO, DAT	1,400	66	12	19	**2.4**	0.9	200	669	921	107	47	Alive
13, M	ITP, AIN	ALPS	1,2	LPR	No	1,300	79	**4**	16	**2**	3.5	**2,150**	430	1,248	630	68	Alive
14, M	ITP, AIN	ALPS	14,9	LPR BMF	ANA, ENA, ASMA, CD	**850**	64	16	19	**2**	**305**	360	725	1,534	376	62	Alive
15, M	ITP, AIN	ALPS LIKE	10	LPR arthralgia	No	1,500	78	19	**2**	**2**	1.3	**640**	0	625	167	42	Alive
16, M	ITP, AIN	ALPS	11,2	LPR	DAT	**780**	69	18	12	**4**	3	**660**	356	820	33	87	Alive
17, M	ITP, AIN	ALPS LIKE	2,2	LPR	DAT	2,600	58	29	11	0	13	**600**	641	694	21	54	Alive
18, M	ITP, AIN	ALPS LIKE	14,8	LPR, recurrent fevers	ENA	**770**	83	**8**	**7**	**4.6**	2	**600**	274	**2,506**	228	174	Alive
19, M	ITP, AIN	ALPS LIKE	14,2	No	No	1,580	80	17	**1.5**	1.3	3	300	484	898	214	158	Alive
20, M	ITP, AIN	ALPS LIKE	7,6	LPR	DAT	1,890	82	12	**3.6**	**2.6**	Na	Na	**1,694**	1,457	36	143	Alive
21, M	AIHA, AIN	ALPS LIKE	6	LPR recurrent fevers, arthralgia, rash	ASMA, ASCA	1,190	73	12	13	**3.4**	Na	275	Na	**2,416**	286	236	Alive
22, F	AIHA, AIN	ALPS	12,6	No	ANA, anti-C3d, anti-dsDNA, SLE, Hashimoto’s thyroiditis	**600**	85	**6**	**7**	**3.9**	3.2	421	655	1,075	205	45	Alive

anti-dsDNA ab, anti-double-stranded DNA antibodies; anti-N ab, anti-neutrophil antibodies; AIHA, autoimmune haemolytic anaemia; AIN, autoimmune neutropenia; ANA, antinuclear antibodies; ANCA, anti-neutrophil cytoplasmic antibodies; ASCA, anti-Saccharomyces cerevisiae antibodies; ASMA, anti-smooth muscle antibodies; B, benign; BMF, bone marrow failure; CD, celiac disease; DAT, direct antiglobulin test; DNT, double-negative T cells; ENA, extractable nuclear antigen antibodies; ITP, immune thrombocytopenia; LAC, lupus anti-coagulant; LPR, lymphoproliferation (chronic, >6 months, non-malignant, non-infectious lymphadenopathy or splenomegaly or both); MAS, macrophage activation syndrome; Na, not available; SLE, systemic lupus erythematosus; Tg, thyroglobulin; TPO, thyroid peroxidase; TTP, thrombotic thrombocytopenic purpura.

In order to see whether there were differences between the patients carrying variants versus those who did not, we divided the whole cohort in these two groups. We did not find any significant difference for any of the tested variables (**Table 5**). However, although not reaching statistical significance, a greater need of second-line therapies was observed in the patients with variants (14/18, 78%) compared with the ones without (14/22, 64%). In line with this, patients carrying variants were also the only ones who required further treatment with stem cell transplantation (SCT): five of them were transplanted from haploidentical (3), sibling (1), and unrelated (1) donor, respectively, and are all alive and well at a median of 4.6 years after the procedure. Two patients (5%) died due to complication of the diseases. The median follow-up was 6.9 years (0,3–35).

**Table 5:** Clinical and immunological differences according to variants.

	Tot (%)	Patients with variants (18/40, 45%)	Patients without variants (22/40, 55%)	p
Females	17/40 (43)	10/18 (56%)	7/22 (32%)	0.13
Familiar history of autoimmunity	9/40 (23)	5/18 (28%)	4/22 (18%)	0.47
Associated immunological features^a^	24/40 (60)	9/18 (50%)	15/22 (68%)	0.22
ALPS phenotype at onset^b^	17/40 (43)	10/18(56%)	7/22 (32%)	0.13
DNT^c^ >1.5%	30/40 (75)	15/18 (83%)	15/22 (68%)	0.27
Signs of lymphoproliferation^d^	27/40 (68)	13/18 (72%)	14/22 (64%)	0.56
Need for second-line therapy	28/40 (70)	14/18 (78%)	14/22 (64%)	0.33
Sequential cytopenia	22/40 (55)	10/18 (56%)	12/22 (55%)	0.94

a Celiac disease, presence of autoantibodies including antinuclear, anti-neutrophils, anti-neutrophil cytoplasmic, anti-smooth muscle, anti-thyroperoxydase, anti-thyeroglobulin, lupus anticoagulant, anti-ADAMTS13, extractable nuclear antigen, cold agglutinins.

b Presence of required and accessory diagnostic criteria for ALPS.

c Double-negative T cells (CD3+ CD4- CD8- TCRαβ+).

d Chronic (> 6 months), non-malignant, non-infectious lymphadenopathy or splenomegaly or both.

## Discussion

Paediatric Evans syndrome is a very rare and challenging disease. To the best of our knowledge, this study reports on the largest monocentric cohort of children, homogenously screened with molecular analysis, and confirms the presence of underlying disorders in a considerable number of cases, highlighting the important role of genetic assessment in this setting of patients. This holds especially true in the case of IEI that are known to have overlapping phenotypes due to the incomplete penetrance of genetic defects and other still unknown epigenetic factors (9, 11).

Previous multicentric studies have already shown the presence of underlying immune dysregulation in most ES patients. However, in these studies, differently from ours in which all patients were genetically tested, patients underwent genetic analysis based on a prescription of the attending physician, on the availability of the diagnostic tools over the years, on financial access to analysis, and on the severity of the disease (6, 14, 15). This might have generated a selected cohort of patients that were more likely to show an underlying defect and might explain the lower detection rate in our patients (48%) compared to that of the larger multicentric French cohort (68%) (15). In addition, our results were obtained using NGS gene panel analysis, thus implying that a deeper investigation with WES or WGS (whole genome sequencing) might have increased the detection rate. Such further analysis should be taken into consideration in patients with negative NGS results, since the presence of other immuno-pathological manifestations in the majority of our patients is in keeping with the idea that both ES and MAC represent an epiphenomenon of some still unknown IEI.

As expected, most of the variants found in our cohort were involved in genes causing CVID, ALPS, or ALPS-like syndromes. Interestingly, patients carrying *CTLA4 *(27) and *DADA2 *(26) variants initially presented with an exclusive haematological phenotype which was followed by more typical signs of their diseases during adolescence/adulthood, highlighting that, in the paediatric age, cytopenia can be the first sign of a more complex disease which can show its complete phenotype later in life. In this respect, the *DADA2* patient’s peripheral cytopenia worsened due to the occurrence of severe bone marrow failure. The coexistence of immune-mediated destruction of blood cells and marrow failure was also shown in both patients (Pt 9 and Pt 16) carrying two very rare variants of unknown significance on the *CARD11* gene, described to be damaging in most scores and also included in the GUK (guanylate kinase-like) domain, critical for protein’s function, where other pathogenic variants have been reported. The phenomenon of the immune-mediated interplay between bone marrow and peripheral blood in the pathogenesis of cytopenia in IEI has already been highlighted by our group (30) and has clinical implications since these patients deserve a particular alert and specific follow-up.

The pathogenic variant *IKBKG* was found in a female presenting with isolated ES during childhood and showing further signs of immune dysregulation during adolescence. The abnormal chromosome X inactivation documented in our female patients carrying a X-linked disorder—known to be the cause of incontinentia pigmenti, ectodermal dysplasia, and immunodeficiency—may explain the milder phenotype. In addition, also the different degree of the protein impairment (NF-kappa-B essential modulator—NEMO), which may depend on the type of variant, may have contributed to the milder clinical issues (31, 32).

As expected, most patients showed higher levels of DNTs, which are well known to be raised not only in patients with ALPS but also in association with ALPS-like or CVID phenotypes (33). This indicates that increased DNTs may represent an important initial screening tool for patients with ES and MAC that can address subsequent specific immunological investigations (4, 34–36).

No clinical and immunological differences were noted comparing patients with or without a genetic diagnosis. Similarly, apart from a slight—although not significant—female predominance in patients with ES and a statistically significant higher presence of immune abnormalities in MAC cases, we did not notice other differences between both groups. This strengthens the concept that multilineage cytopenia can be an epiphenomenon of an underlying disorder, regardless of the involved cell line. Nonetheless, the several additional manifestations of autoimmunity and of immune dysregulation noted in patients with MAC may reflect the more heterogeneous genetic background we found in this group.

As already reported in the French cohort (15), most patients needed second- or further-line immunosuppressive therapies which, in most cases, were successful. In fact, treatments as mycophenolate mofetil and sirolimus, well known to be effective in autoimmune cytopenias (37, 38), represent an appropriate approach for patients non-responding to steroids. Nonetheless, the identification of specific molecular defects, following a proper genetic screening, may lead to the administration of targeted therapies, as in the case of one patient of our cohort, affected with *CTLA4* haploinsufficiency, who was successfully treated with abatacept (27). Few non-responding patients—all with a demonstrated underlying defect—successfully underwent SCT (39).

In conclusion, both ES and MAC should be considered an epiphenomenon of underlying IEI which are detected in about half of patients. Therefore, in these cases, genetic screening has to be considered a fundamental step of the diagnostic work-up that should be offered to all patients who may potentially benefit from specific follow-up and treatments.

## Data Availability Statement

The datasets presented in this study can be found in online repositories. The names of the repository/repositories and accession number(s) can be found as follows: https://www.ncbi.nlm.nih.gov/clinvar/, SCV001424053-SCV001424130.

## Ethics Statement

The studies involving human participants were reviewed and approved by Comitato etico Regione Liguria. Written informed consent to participate in this study was provided by the participants’ legal guardian/next of kin.

## Author Contributions

MM and DG designed the research and wrote the paper. AG, MaL, MT, and IC performed the genetic analysis. PT, EC, and MiL performed the laboratory assays and functional studies. EP, FF, GO, and AB contributed the clinical data. CD coordinated the research and revised the manuscript. All authors contributed to the article and approved the submitted version.

## Funding

We acknowledge ERG S.p.A., Rimorchiatori Riuniti (Genoa), Cambiaso Risso Marine (Genoa), Saar Depositi Oleari Portuali (Genoa), ONLUS Nicola Ferrari, and Ministero della Salute-Ricerca corrente 2021 for supporting the activity of Hematology Unit of IRCCS Istituto Giannina Gaslini.

## Conflict of Interest

The authors declare that the research was conducted in the absence of any commercial or financial relationships that could be construed as a potential conflict of interest.

## Publisher’s Note

All claims expressed in this article are solely those of the authors and do not necessarily represent those of their affiliated organizations, or those of the publisher, the editors and the reviewers. Any product that may be evaluated in this article, or claim that may be made by its manufacturer, is not guaranteed or endorsed by the publisher.
