# Supplementary material for: Underlying Inborn Errors of Immunity in Patients With Evans Syndrome and Multilineage Cytopenias: A Single-Centre Analysis
Source: Front Immunol. 2022 May 17;13:869033. doi: 10.3389/fimmu.2022.869033 (PMC9152001; doi:10.3389/fimmu.2022.869033)
Supplement: Supplementary file 1 [file Table_1.pdf]

Gene	RefSeq NM_	Coding position	Protein
FAS	NM_000043.6	c.766G>C	p.Glu256Gln
FAS	NM_000043.6	c.819G>T	p.Gln273His
CTLA4	NM_005214.4	c.168_171delTGTG	p.Cys58Sfs*13
IKBKG	NM_001099856.6	c.373G>A	p.Glu125Lys
CARD11	NM_032415.7	c.2899C>T	p.Arg967Cys
ADA2	NM_177405.3	c.559A>C	p.Thr187Pro
ADA2	NM_177405.3	c.563T>C	p.Leu188Pro
STAT3	NM_213662.2	c.1973A>G	p.Lys658Arg
LIG4	NM_001098268.2	c.833G>A	p.Arg278His homo
LRBA	NM_001367550.1	c.1963C>T	p.Arg655Ter homo
FAS	NM_000043.6	c.385T>C	p.Cys129Arg
STAT3	NM_001369512.1	c.454C>T	p.Arg152Trp
CARD11	NM_032415.7	c.3025G>A	p.Val1009Ile
RAG1	NM_001377280.1	c.1520G>A	p.Arg507Gln homo
TNFRSF13B	NM_012452.3	c.581_582delCCinsAA	p.Ser194Ter
TNFRSF13B	NM_012452.3	c.605G>A	p.Arg202His
TNFRSF13B	NM_012452.3	c.171G>C	p.Gln57His
CASP10	NM_032977.4	c.1228G>A	p.Val410Ile

Coding positions of the found variants.
